# Correction: A comprehensive approach for elucidating the interplay between 4f^*n*+1^ and 4f^*n*^5d^1^ configurations in Ln^2+^ complexes

**DOI:** 10.1039/d5sc90097b

**Published:** 2025-05-07

**Authors:** Maria J. Beltran-Leiva, William N. G. Moore, Tener F. Jenkins, William J. Evans, Thomas E. Albrecht, Cristian Celis-Barros

**Affiliations:** a Theoretical Division, Los Alamos National Laboratory Los Alamos New Mexico 87545 USA; b Department of Chemistry, University of California Irvine California 92697-2025 USA; c Radioisotope Science and Technology Division, Oak Ridge National Laboratory Oak Ridge TN 37830 USA celisbarroca@ornl.gov; d Department of Chemistry, Nuclear Science & Engineering Center, Colorado School of Mines Golden Colorado 80401 USA

## Abstract

Correction for ‘A comprehensive approach for elucidating the interplay between 4f^*n*+1^ and 4f^*n*^5d^1^ configurations in Ln^2+^ complexes’ by Maria J. Beltran-Leiva *et al.*, *Chem. Sci*., 2025, **16**, 2024–2033, https://doi.org/10.1039/d4sc05438e.

The original version of this manuscript contains an error in the caption for Table 1 as the incorrect computational method was referenced. The methodology given in the caption should be CASSCF/SO, rather than MC-pDFT.

The full corrected caption is as follows and replaces the original caption within the manuscript:

**Table 1** Ground state Natural Spin Orbital (NSO) occupations for Ln^3+^ free ions, LnCp_3_ and [LnCp_3_]^−^ complexes from the CASSCF/SO calculations. The total angular momentum quantum number (*J*) along with its predominant M_*j*_ are specified. In Fig. S5 a depiction of the f-orbitals and their labels are shown.

The Royal Society of Chemistry apologises for these errors and any consequent inconvenience to authors and readers.

